# Hypofractionated Radiotherapy in Gynecologic Malignancies—A Peek into the Upcoming Evidence

**DOI:** 10.3390/cancers16020362

**Published:** 2024-01-15

**Authors:** Razan Amjad, Nataliya Moldovan, Hamid Raziee, Eric Leung, David D’Souza, Lucas C. Mendez

**Affiliations:** 1 Department of Radiation Oncology, King Abdulaziz University, Rabigh 25732, Saudi Arabia; 2 Department of Radiation Oncology, London Health Sciences Centre, London, ON N6A 5W9, Canada; 3 Department of Radiation Oncology, BC Cancer, Kelowna, BC V1Y 5L3, Canada; 4 Department of Radiation Oncology, Sunnybrook Health Sciences Centre, Toronto, ON M4N 3M5, Canada

**Keywords:** hypofractionated radiotherapy, gynecologic malignancies, cervical cancer, uterine cancer, universal access to radiotherapy

## Abstract

**Simple Summary:**

Radiotherapy (RT) is often part of the curative intent treatment in gynecologic oncology. In cervical and uterine cancers, RT is typically delivered daily over five weeks. Recently, advances in technology have allowed for higher doses of RT to be given per fraction with an overall shorter treatment time. This treatment course, called hypofractionated RT, has become the standard of care in other pelvic sites such as the prostate and rectum and is being investigated in gynecologic malignancies. In addition, hypofractionation offers a potential solution in low-resource settings where there is insufficient access to radiotherapy as with the challenges faced during the COVID-19 pandemic worldwide. This review summarizes the rationale and application for hypofractionation, the available literature and ongoing clinical trials in the gynecologic space.

**Abstract:**

Radiotherapy (RT) has a fundamental role in the treatment of gynecologic malignancies, including cervical and uterine cancers. Hypofractionated RT has gained popularity in many cancer sites, boosted by technological advances in treatment delivery and image verification. Hypofractionated RT uptake was intensified during the COVID-19 pandemic and has the potential to improve universal access to radiotherapy worldwide, especially in low-resource settings. This review summarizes the rationale, the current challenges and investigation efforts, together with the recent developments associated with hypofractionated RT in gynecologic malignancies. A comprehensive search was undertaken using multiple databases and ongoing trial registries. In the definitive radiotherapy setting for cervical cancers, there are several ongoing clinical trials from Canada, Mexico, Iran, the Philippines and Thailand investigating the role of a moderate hypofractionated external beam RT regimen in the low-risk locally advanced population. Likewise, there are ongoing ultra and moderate hypofractionated RT trials in the uterine cancer setting. One Canadian prospective trial of stereotactic hypofractionated adjuvant RT for uterine cancer patients suggested a good tolerance to this treatment strategy in the acute setting, with a follow-up trial currently randomizing patients between conventional fractionation and the hypofractionated dose regimen delivered in the former trial. Although not yet ready for prime-time use, hypofractionated RT could be a potential solution to several challenges that limit access to and the utilization of radiotherapy for gynecologic cancer patients worldwide.

## 1. Introduction

Radiotherapy (RT) has a fundamental role in the treatment of gynecologic malignancies. In cervical cancers, radiotherapy is offered as a curative option for locally advanced tumors. Meanwhile, for uterine cancers, it is mostly being utilized in the adjuvant setting with the goal of improving locoregional control. Other gynecologic malignancies treated with definitive radiotherapy include vaginal and vulvar cancers, which are both rare entities [1,2]. Given the rarity and pathology of vaginal cancers, the radiotherapy data for vaginal cancers are typically extrapolated from cervical cancer trials [3]. In vulvar cancers, RT is offered as a radical treatment to non-surgical patients or in scenarios in which surgery is too morbid. Radical RT, however, has a significant side effect profile, and moderate to severe mucocutaneous reactions are frequently seen in clinical practice [4]. The role of radiation in ovarian cancers is typically palliative [5,6,7].

Hypofractionated radiotherapy is an alternative fractionation scheme that delivers higher doses of radiation in fewer fractions compared to conventional fractionation. Hypofractionated radiation therapy has gained popularity and is already a standard-of-care option in some sites, including but not limited to prostate [8,9,10,11], breast [12,13,14,15,16] and rectal [17,18,19,20] cancers. In the gynecologic oncology setting, the role of hypofractionated radiotherapy is still not well understood. It is the subject of investigation for multiple efforts in cervical and uterine cancers, as described below. Hypofractionation in vaginal cancers may follow the evidence in cervical cancer patients [3], while the expected higher acute toxicity to skin and mucosa would likely preclude its use in radical vulvar cancer treatments [4]. The uptake of hypofractionated RT in different sites is driven by different factors including improvements in technology, the search for more cost-effective treatment, a better understanding of cancer radiobiology and, more recently, the COVID-19 pandemic [21,22,23,24].

Technological advances in radiotherapy including intensity-modulated RT (IMRT) and volumetric arc radiotherapy (VMAT) result in better dose shaping and conformality, reducing delivery of the prescribed dose to the surrounding organs at risk (OARs) [25,26]. In the gynecologic setting, RTOG 1203 demonstrated that IMRT reduces patient toxicities such as diarrhea, and has a less detrimental impact on genitourinary and gastrointestinal outcomes in the post-operative setting when compared to conformal radiotherapy (CRT) techniques [27]. A similar randomized trial of post-operative radiation in cervical cancer patients comparing IMRT and 3-D CRT titled PARCER showed a reduction in grade 2 or higher late gastrointestinal toxicity from 42% to 21% with the use of IMRT [28]. Image-guided radiotherapy (IGRT) is another technological development that is synergic to IMRT since it allows for accurate soft tissue visualization during RT delivery, leading to smaller error setup margins and a smaller overall target volume [29,30]. Moreover, IGRT also allows for anatomical verification seconds prior to beam delivery, preventing RT delivery in conditions such as non-reproducible bladder/rectal filling. Ultimately, the utilization of these combined technologies results in a more controllable dose delivery environment and a better understanding of the relationship between dose, volume and OAR toxicity. This has facilitated a safe increase in dose per fraction and a reduction in the total number of fractions, resulting in hypofractionationated radiotherapy regimens [24,27,29].

Despite radiotherapy being one of the fundamental oncologic treatments, there are gaps in access to RT worldwide. Different metrics are available to estimate radiotherapy access, including the number of radiotherapy services per capita. A wide range of RT availability is seen worldwide, with greater than 92% in Europe to just 34% in Africa, while being approximately 200% in North America [31]. Non-universal access to radiotherapy is a pressing concern, particularly when it comes to treating cervical cancer, a disease that affects hundreds of thousands of women globally. Cervical cancer is one of the most preventable and curable cancers when detected early and treated appropriately, with radiotherapy playing a pivotal role in its management in curative and palliative settings [32]. However, the reality is that not all individuals around the world have equal access to this life-saving treatment modality. Importantly, many of the countries that have the highest incidence of cervical cancer have the most limited access to radiotherapy [33,34,35,36]. Disparities in access to radiotherapy for cervical cancer are multifaceted and can be attributed to an interplay of economic, geographic, social and healthcare system factors [37]. Moreover, insufficient access to radiotherapy has a substantial negative impact on cancer survival [38,39]. In the Brazilian public health system, for instance, more than 5000 deaths could be prevented in the most prevalent cancer types if access to radiotherapy was universal [40].

The hypofractionation of lengthy radiotherapy courses, where the equivalent radiation dose is delivered in fewer fractions, has several implications for patients and the healthcare system, including decreased resource utilization with fewer human resources and less radiotherapy machine time needed, increased patient throughput and less cost and time for patients [41,42,43]. In addition, hypofractionated RT can address the challenge of non-universal RT access and provide comprehensive cancer care, resulting in better quality of life and cancer survival [40,44,45,46].

Hypofractionated radiotherapy has several potential advantages over conventional fractionation, including (1) decreasing resource utilization and the cost to the healthcare system [42,44,47]. Hypofractionation reduces the overall treatment machine utilization time, allowing healthcare facilities to treat more patients using similar machine resources [23,42]. In systems already offering universal radiotherapy access, hypofractionated treatment has the potential to reduce the overall costs through less personnel utilization, thereby improving treatment’s cost efficiency. (2) There is less cost to patients, as fewer treatment fractions results in fewer hospital visits and minimized expected associated costs with transportation, accommodation and time off work for both patients and their caregivers [41,44,45]. (3) Finally, shorter treatment regimens may alleviate some of the logistical challenges associated with coordinating combined therapies and prolonged therapy schedules. For example, adjuvant treatment for uterine cancer may include sequential systemic therapy and radiotherapy/chemoradiotherapy or a “sandwich” approach where RT is delivered between chemotherapy cycles [48,49,50].

**Figure 1:**
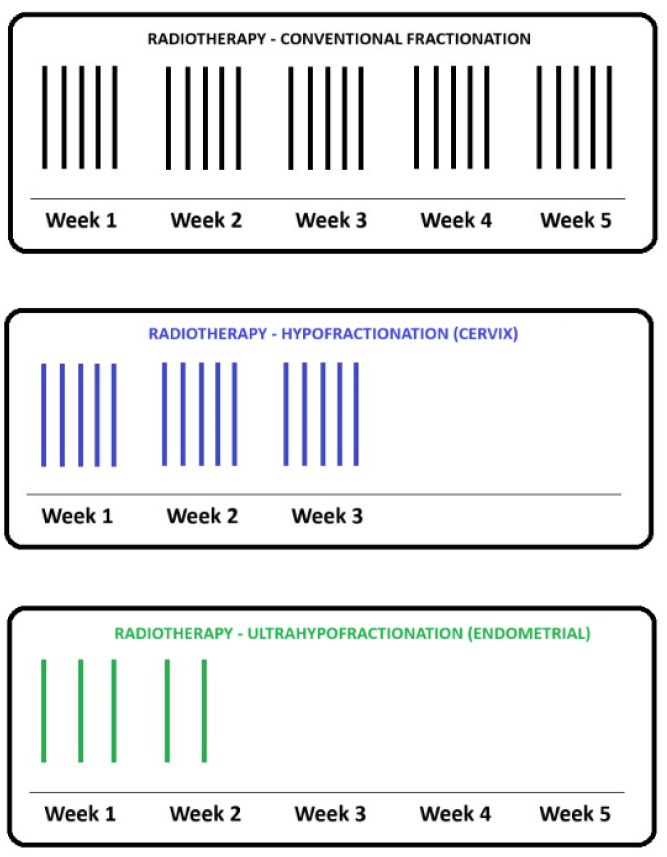
Schema of a typical external beam radiotherapy (EBRT) course using conventional fractionation in gynecologic malignancies with 25 fractions delivered daily over 5 weeks (**top**, in black). This fractionation is typically seen in radical EBRT treatments of locally advanced cervical cancers or in the adjuvant setting of endometrial cancer, post-hysterectomy. The role of a moderately hypofractionated course with 15 daily fractions is under investigation in randomized phase 2 trials involving cervical cancer patients (**middle**, in blue). The role of ultrafractionated regimes with five fractions delivered every other day is currently being investigated in a phase 2 randomized trial in the adjuvant endometrial cancer setting (**bottom**, in green).

Figure 1 provides examples of the hypofractionated schemes that are being actively investigated in randomized clinical trials in gynecologic malignancies. The typical radical and adjuvant radiotherapy courses used in cervical and uterine cancers, respectively, are 5 weeks of radiation delivered daily, Monday to Friday with weekends and holidays off [21,51]. In addition, there are weekly clinical appointments for side effect monitoring in patient review clinics. From a patient’s perspective, this is 25 or more appointments to attend. For patients who do not live near a cancer center with radiotherapy, this either means lengthy daily commutes or staying away from home for the duration of treatment. This results in decreased quality of life for patients and additional financial and emotional stressors [43,52]. A hypofractionated radiotherapy course of up to 3 weeks in duration reduces the patient’s burden and cost as it is shorter than the current standard of care [42]. In addition, each radiation fraction delivered requires radiation therapists, as well as nursing, clerical and physician support, which are all human resources utilized at the cancer center. With a shorter hypofractionated course of radiotherapy, the patient throughput can be increased for the same machine time and human resources [46].

More recently, the concept of cost efficiency had particular relevance in the backdrop of the COVID-19 pandemic since it resulted in an unprecedented strain on healthcare systems worldwide. This led to disruptions in cancer care services such as cancer screening, the referral of symptomatic patients, diagnosis and definitive treatments [53,54,55,56,57]. The COVID-19 pandemic may have had a greater impact on gynecologic cancer patients stemming from the complex nature of their clinical care and various socioeconomic factors [53,58]. A survey of practicing gynecologic radiation oncologists in the United States reported that a significant proportion of the surveyed individuals experienced a temporary suspension in the surgical management of gynecologic patients for from one to three months; the transition to telemedicine as the primary mode of patient care and the use of shorter brachytherapy schedules as aftermath of the pandemic [59]. Equally, 45 percent of respondents reported a treatment interruption or delay resulting from COVID-19 positivity, while 55% indicated that patients themselves opted to postpone their care due to concerns related to COVID-19 [59]. In India, the real-world compliance to radiation treatment in gynecologic cancers during the COVID-19 pandemic had a significant decline, with 76% of patients completing radiotherapy and only 26% receiving the full course of concurrent chemotherapy [57]. In 72% of patients there was a treatment delay due to COVID-19 infection or the logistics caused by COVID-19, with the resulting overall treatment time greater than 56 days [57]. The detrimental effects of the COVID-19 pandemic on cancer care delivery were especially concerning in cervical cancer patients given the pivotal role RT plays in this setting and the known worse outcomes associated with a longer overall treatment time [60]. With this in mind, several recommendations in gynecologic oncology were published involving hypofractionated radiotherapy [21] and patient treatment prioritization as a guidance tool for systems strained by the COVID-19 pandemic [61,62,63].

Another reason for the current interest in hypofractionation relates to new clinical evidence suggesting that repopulation plays an important role in total biological dose, a factor that has been overlooked for quite some time in gynecology oncology. Classically, biologic equivalent doses (BEDs) have been estimated by taking into consideration the tissue α/β ratio, the dose per fraction and the total dose, with little reference to the total time for treatment completion [64]. With the use of this simplified formula, hypofractionated RT to cancers with high α/β ratios are likely futile as higher doses per fraction would increase the biological doses to the surrounding OARs. This would consecutively lead to higher rates of long-term toxicity due to the low α/β ratio of the normal tissues in relationship to the cancer cells with a high α/β ratio [65]. However, a plethora of clinical data seems to challenge this paradigm.

In the United Kingdom, two phase 3 trials (BC2001 and BCON) randomized patients with muscle invasive bladder cancer between radiotherapy alone or radiotherapy with a radiosensitizer (either 5-fluorouracil and Mitomycin or Carbogen Nicotinamide). In both trials, two different radiotherapy fractionations were allowed: 64 Gy in 32 fractions or 55 Gy in 20 fractions, with the offered regimen decided by the physician involved in the case. Interestingly, despite having a reduced estimated BED (76.8 Gy_10_ vs. 70.1 Gy_10_), patients undergoing hypofractionated radiotherapy had higher tumor control [66] than patients receiving 2 Gy per fraction. In corroboration, the results from the recently presented HYPNO phase 3 clinical trial involving patients with locally advanced head and neck cancers demonstrate that 55 Gy in 20 fractions is non-inferior to 66 Gy in 33 fractions in terms of cancer control, despite the lower estimated BED using the traditional formula [67]. In cervical cancers, the overall treatment time has been shown to correlate with cancer control, with treatments delivered in less than 50 days resulting in higher rates of a complete response [68]. Therefore, it seems plausible to hypothesize that repopulation plays a key role in the total biological dose of a given radiation treatment in cervical cancers, as seen in bladder and head and neck tumors. Thus, the shorter overall treatment time may compensate for a slightly reduced predicted BED using the conventional formula associated with hypofractionated RT to tissues with high α/β ratios.

In summary, there are multiple potential merits of hypofractionated radiation therapy in gynecologic oncology. Hypofractionated radiotherapy is a standard-of-care option in other pelvic malignancies such as prostate and rectal cancers and has helped different institutions worldwide to address the temporary challenges posed by the COVID-19 pandemic [21]. While the COVID-19 pandemic is now subsiding, there are ongoing challenges in radiotherapy access worldwide [31,40,44]. This treatment regimen has the potential to permanently improve global access to radiation therapy without compromising care at an individual patient level, especially if hypofractionation is shown to perform as well as conventional radiotherapy treatments in terms of toxicity. The evidence for hypofractionation in gynecologic malignancies is still evolving and there is a currently a paucity of literature on this subject. This review summarizes the rationale, challenges and recent developments associated with hypofractionated RT in gynecologic malignancies, focusing on cervical and uterine cancers.

## 2. Methods

A comprehensive search for published clinical data on and ongoing clinical trials of hypofractionated radiotherapy in uterine and cervical cancer was performed. The following databases were searched—PubMed, Google Scholar and Semantic Scholar—to access retrospective, prospective and randomized clinical trials. Abstract proceedings of relevant meetings for the American Society for Radiation Oncology (ASTRO) were also included. Ongoing clinical trials were identified using ClinicalTrials.gov. The exclusion criteria were palliative intent radiotherapy, stage IV gynecologic malignancy and adjuvant radiotherapy in cervical cancer patients. All searches were updated until 11 October 2023.

## 3. Hypofractionation in Cervical Cancer

Cervical cancer is a major cause of morbidity and mortality worldwide with radiation therapy being a fundamental component of treatment [33]. In the locally advanced setting, radiotherapy for five weeks with concurrent chemotherapy followed by a brachytherapy boost has been the standard-of-care treatment for multiple decades. However, alternative regimens have been poorly investigated, with only a few studies exploring the use of hypofractionated RT in the treatment of cervical cancer.

In a study conducted in Brazil, researchers evaluated the role of hypofractionated RT administered with a four-field box technique to the whole pelvis and a total dose of 40 Gy with 2.5 Gy fractions delivered twice a day on specific days [69]. A low-dose-rate brachytherapy boost of 35 Gy prescribed to point A in a single insertion was given on day 29. The total overall radiotherapy time was 61 days. This treatment approach was demonstrated to be well tolerated with no grade 4 toxicities. In terms of efficacy, the study found that the hypofractionated RT regimen resulted in a high rate of disease control with a complete response rate of 85% and a 5-year overall survival of 59%. In another series investigating hypofractionated radiotherapy, researchers from South Africa conducted a study with 104 patients with stage IIIB cervical cancer who were treated with external beam radiotherapy (EBRT) using 40 Gy in 16 daily fractions followed by brachytherapy consisting of 9 Gy in 2 fractions [70]. In this retrospective cohort, 70% of patients achieved a complete response, and the disease-free survival at 20 months was 59%. Importantly, five patients developed late gastrointestinal toxicity—one patient had grade 2 toxicity and four patients had grade 3 toxicity of radiation proctitis. There were no late genitourinary toxicities observed. A third retrospective report evaluating the role of hypofractionated RT comes from Tata Memorial Hospital in India. In this study, 62 patients with stage IIIB cervical cancer were treated with 39 Gy in 13 daily fractions using mostly a two-field technique with AP/PA fields followed by brachytherapy [71]. The 5-year disease-free survival rate was 59% and five patients developed late grade 3 rectal toxicity.

Overall, these reports seem to support moderate hypofractionated RT with a total dose around 39–40 Gy being well tolerated in patients with locally advanced cervical cancer, despite the use of older radiotherapy techniques. However, there is significant heterogeneity between these studies, with a lack of group control or rigorous toxicity analysis. This makes it challenging to draw conclusions related to the efficacy associated with the regimens investigated in these series. Moreover, the RT techniques used are known to be inferior in terms of toxicity and cancer control compared to modern techniques involving IMRT, IGRT and image-guided brachytherapy. Thus, evidence on the role of modern hypofractionated RT is scarce, and further research is required in this space.

Several ongoing clinical trials are currently investigating the role of hypofractionated RT in cervical cancer treatment around the globe [72,73,74,75,76,77]. In India, a single-arm prospective study with 50 patients with stage IB-IIIC1 squamous cell carcinoma of the cervix treated with EBRT, consisting of 40 Gy in 16 fractions, has been preliminary reported in abstract format, with encouraging tolerability results [72]. Patients were treated using 3D CRT with concurrent cisplatin and a high-dose-rate brachytherapy boost of 28 Gy in four fractions. Equally, 6 out of 50 patients also received a sequential pelvic lymph node boost consisting of 10 Gy in four fractions. Acute grade 2 and grade 3 gastrointestinal toxicity was observed in 20 (40%) and 10 (20%) of the patients, respectively, and acute grade 2 and 3 genitourinary toxicity in 5 (10%) and 3 (6%) patients. In terms of late toxicity, the authors report late grade 2 gastrointestinal toxicity in six (12%) patients and two (4%) patients with late grade 3 gastrointestinal toxicity. Three patients (6%) experienced late grade 2 genitourinary toxicity. No grade 3 genitourinary toxicity was reported. The 3-year overall survival was 90.6% and 3-year disease-free survival was 92.7% with three patients developing recurrence and succumbing to their disease.

In Mexico, a phase II randomized trial comparing the safety and response rate between standard versus hypofractionated RT treatment regimens in patients with clinical stage III cervical cancer is open [74]. This trial aims to enroll 82 patients and randomize them between the standard fractionation of 45 Gy in 25 fractions and hypofractionated RT of 37.5 Gy in 15 fractions. Patients will be treated with four-field box EBRT with weekly cisplatin followed by a brachytherapy boost. The primary endpoint of the trial is acute and late toxicity as evaluated using version 4.03 of CTCAE and RTOG scoring system. Of note, this trial is offering RT with conformal techniques while assessing the toxicity outcomes, which could be higher when compared to techniques involving modulated radiotherapy [27].

In another trial, researchers from Iran are investigating whether hypofractionated chemoradiation is non-inferior to standard treatment in terms of the clinical response and toxicity in patients with cervical cancer [75]. The trial aims to enroll 60 patients with cervical cancer stages IB to IIIC and randomize them between the standard treatment of 45 Gy in 25 fractions and hypofractionation RT with a total dose of 40 Gy in 15 daily fractions. The co-primary endpoints are early toxicity and early response. Radiotherapy will be delivered concurrently with weekly cisplatin and followed by a brachytherapy boost of 28 Gy in four fractions, similar to the Mexican study [74].

A third ongoing clinical trial comes from the Philippines (entitled HYACINCT) and is a single-arm trial with a two-phase design [76]. This study evaluates the safety and effectiveness of hypofractionated RT in chemotherapy-ineligible patients diagnosed with locally advanced cervical cancer. The first phase of this trial is a dose escalation evaluation of the maximum tolerated dose to the gross lymph node(s) in the use of simultaneous integrated boost (SIB) and intensity-modulated radiotherapy. In the second phase of this trial, the clinical response of hypofractionated RT with or without nodal SIB is evaluated using a single-arm design. The hypofractionated RT regimen will consist of 40 Gy in 15 daily fractions to the whole pelvis with nodal SIB 45–48 Gy followed by four fractions of a brachytherapy boost at 6.5–7.5 Gy per fraction. Unlike the ongoing trials in Mexico and Iran, this study has a different design, and is the only trial known to us that is specifically looking at the maximum tolerated SIB dose to the lymph nodes [74,75,76].

HYPOCx-iRex is a Thai phase II randomized trial in locally advanced cervical cancer, comparing hypofractionated RT of 44 Gy in 20 daily fractions using IMRT/VMAT to the whole pelvis and 53 Gy in 20 fractions SIB to the gross lymph nodes with weekly cisplatin with the standard treatment of 45 Gy in 25 daily fractions to the whole pelvis and 55 Gy SIB to the gross lymph nodes with weekly cisplatin [77]. The planning aims for both EBRT and image-guided adaptive brachytherapy (IGABT) were adapted from EMBRACE2 in this trial. An interim analysis reporting on late toxicity, the oncologic outcome and the pattern of failure at 6 months post-radiation was recently presented at ASTRO 2023 [77]. The presented data included 29 patients with a median follow-up time of 8 months from the start of RT. There was no statistically significant difference in the dose delivered to the high-risk CTV (HRCTV) at the time of IGBT (EQD2 D90 of 90 Gy and 89.8 Gy in the hypofractionated arm and standard arm, respectively). The authors report one patient with grade 3 crude gastrointestinal toxicity in the hypofractionated arm, but no grade 3 late and persistent (LAPER) GI side effects in both arms. There was no grade 3 crude genitourinary toxicity noted. In this short-term analysis, there was no significant difference in oncologic outcome and pattern of failure [77].

**Figure 2:**
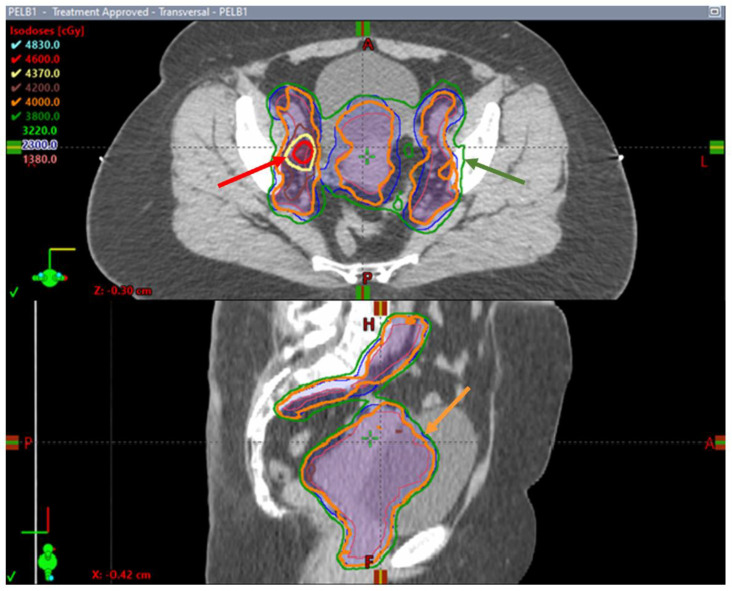
Axial and sagittal images of a hypofractionated treatment plan delivered to a patient enrolled in the HEROICC clinical trial. Note the simultaneous integrated boost (SIB) to the right external iliac enlarged lymph node with a prescription dose of 46 Gy outlined in red (red arrow) while the remaining pelvis is covered by the 95% prescription isodose line (relative to 40 Gy) outlined in green (green arrow). The prescription dose of 40 Gy runs tightly around CTVs (orange arrow).

In Canada, the Hypofractionated External Beam Radiotherapy for Intact Cervical Cancer (HEROICC) trial is a multicentric phase II randomized trial of hypofractionated EBRT versus standard radiotherapy arms [73]. The trial is currently active and enrolling cervical cancer patients with low-risk locally advanced primary disease and limited nodal involvement. Patients with FIGO stages IA to IIB are candidates if they are not considered to be surgical candidates. Also, patients with FIGO stage IIIC1 disease and no common iliac nodal disease, pelvic lymph nodes (<3 cm in largest dimension) and less than three pathologic nodes are candidates for this trial. Radiotherapy is given using VMAT to a dose of 40 Gy in 15 daily fractions in the experimental arm and 45 Gy in 25 daily fractions in the standard arm, both with concurrent weekly cisplatin followed by an image-guided brachytherapy boost. SIB is mandated to the abnormal nodes with doses of 46–48 Gy in the hypofractionated arm (Figure 2) and 55–57.5 Gy in the conventional arm. This trial investigates the feasibility of patient enrollment in the Canadian healthcare system, although multiple secondary outcomes are of interest, including tumor downstaging on magnetic resonance (MR) imaging at the time of brachytherapy; bowel, urinary and sexual quality of life measured as using PRO questionnaires and survival endpoints, including locoregional progression-free survival and metastasis-free survival [73].

Brachytherapy is a crucial part of treatment for locally advanced cervical cancer and is associated with improved cancer survival outcomes when compared to other boost modalities [78]. Brachytherapy delivers approximately 40–50% of the biological dose to the primary gross disease and cervix while minimizing radiation exposure to the organs at risk that surround the target. Typically, regimens involving 4–6 fractions of brachytherapy are recommended by the guidelines [79], although some reports have assessed the role of a brachytherapy boost in 3 or less fractions [80,81,82]. In a large randomized multicentric IAEA trial, FIGO stage IIB and IIIB cervical cancer patients were randomized to 7 Gy × 4 fractions or 9 Gy × 2 fractions post-EBRT to the pelvis [83]. The brachytherapy dose was prescribed to point A in the use of the conventional technique. Not surprisingly (considering the difference in BED between regimens, 39.7 vs. 28.5 EQD2Gy_10_), the 5-year higher local control rate (88% vs. 78%) favored the four-fraction arm, suggesting that the experimental fractionation should not be utilized in clinical practice [83].

Although the IAEA trial is the only randomized study in cervical brachytherapy hypofractionation, there are three retrospective series looking at the outcomes with a three-fraction high-dose-rate brachytherapy boost [80,81,82]. A recent study from the United States compared stage IA2-IVA cervical cancer patients who received 24 Gy in 3 fractions versus 28–30 Gy in 4–5 fractions with the primary outcome being 2-year local failure, as well as survival and toxicity outcomes [82]. There were 32 patients in the three-fraction group and 118 patients in the longer fractionation group with a median follow-up time of 22 months. There was no statistically significant difference in local control between the two groups, with a 2-year local failure rate of 3.6% in the 3-fraction group versus 7.5% with 4–5 fractions. There was also no statistically significant difference in overall survival, disease-free-survival and distant metastasis. Grade 2 toxicity outcomes were reported in 3 of 32 (9.4%) patients in the three-fraction group and 9 of 118 (7.6%) in the longer fractionation group but the genitourinary or gastrointestinal symptoms were not specified. There was also grade 3 toxicity in 2 of 32 (6.3%) versus 7 of 118 (5.9%) patients in the 3-fraction versus 4–5-fraction groups, respectively, and these were not statistically different. The authors also reviewed hospitalization records, looking at serious adverse events, with no difference registered [82].

Two Canadian cohorts looking at brachytherapy boost in a three-fraction regimen of 8 Gy per fraction have also shed some light in the field of hypofractionated brachytherapy [80,81]. In a study from Montreal, 282 patients were retrospectively evaluated with stage IB to IVA cervical cancer treated with 45 Gy in 25 fractions to the pelvis and 24 Gy in 3 fractions of brachytherapy boost prescribed to point A [80]. No chemotherapy was administered to these patients treated in the 1980s and 1990s. The overall genitourinary and gastrointestinal toxicity at 15 years was 8% and 15%, respectively. Of note, there was RTOG/EORTC grade 4 gastrointestinal and genitourinary toxicity reported in 5% and 0.5% of the patients, respectively. The local failure rate at 15 years in this patient cohort overall was 14.5%. The authors concluded that three-fraction brachytherapy regimen is well tolerated and resulted in comparable outcomes to fractionations with larger number of fractions [80]. In the era of image-guided radiotherapy, a dosimetric analysis of MRI-guided brachytherapy plans between three and four fractions was reported from investigators in Toronto [81]. In this study, 224 patients with FIGO stage IB-IVA cervical cancer were retrospectively reviewed. The results showed that patients treated with 24 Gy in three fractions had comparable GTV and HRCTV doses and lower doses to the organs at risk when compared to patients treated with 28 Gy in four fractions. Of note, HRCTVs were statistically smaller in the three-fraction cohort [81].

In summary, the studies using hypofractionated brachytherapy regimens indicate favourable dosimetry, good tolerability and safety using at least three fractions [80,81,82]. However, the data are retrospective in nature and non-comparative. Thus, caution in use and continued monitoring is recommended.

## 4. Hypofractionation in Uterine Cancer

Among all gynecologic malignancies, uterine cancer is the most common gynecologic cancer in North America [33], and an illness primarily treated with surgery. The role of radiation therapy is typically adjuvant with the goal of reducing locoregional recurrence [51]. Postoperative pelvic radiotherapy targets the upper vagina, paravaginal tissue, parametria and pelvic lymph nodes with an overall dose of approximately 45–50 Gy delivered in daily fractions over 5 weeks [51]. However, adjuvant radiotherapy for uterine cancers could be burdensome for patients in terms of the total length of treatment and associated travel time and cost, which may negatively affect patients’ experience [52,84]. In addition, there is significant healthcare resource utilization when standard treatments are delivered daily for a total of 5 weeks [84]. The challenges associated with adjuvant treatment sequencing between radiotherapy and systemic therapy are also an issue [85]. In this setting, hypofractionated radiotherapy is seen as a potential solution, and there are mounting data investigating its role in this space [86].

**Figure 3:**
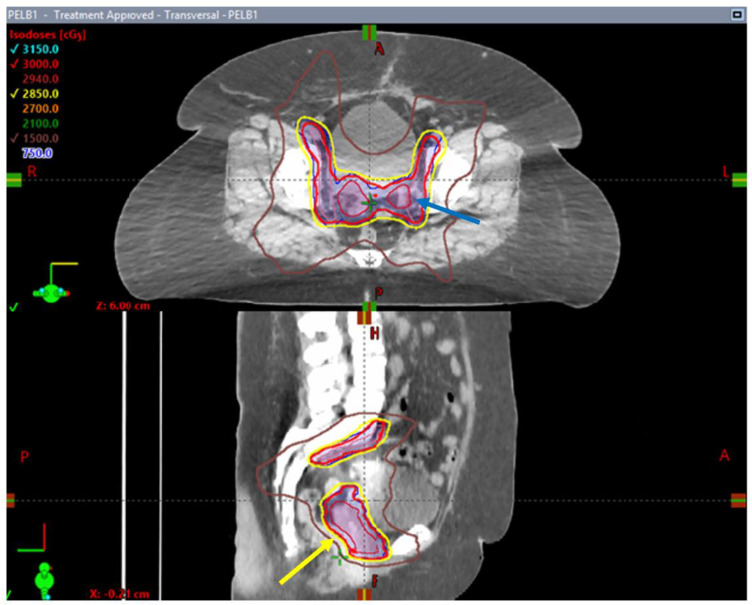
Axial and sagittal images of an SBRT treatment plan delivered to a patient enrolled in the SPARTACUS clinical trial. Note the conformality of the 95% prescription isodose line (yellow arrow) outlined in yellow around PTV volumes (in dark blue) and the homogenous dose distribution within treated volumes with absence of pockets of 105% isodose lines (blue arrow).

In Canada, researchers looked at the role of stereotactic body radiotherapy as an adjuvant therapy post-surgery. SPARTACUS was conceived as a phase I/II, single-arm, multi-center trial assessing the safety and tolerance associated with SBRT in this setting [86]. This single-arm design trial was open in two centers in Ontario and included 61 patients with uterine cancer stages I–III. Adjuvant SBRT to the pelvis was administered every other day or once weekly to a dose of 30 Gy in five fractions (Figure 3). With a median follow-up time of 9 months, the early toxicity results indicated that hypofractionated RT was well tolerated, with 41% of the patients presenting grade 1 and only 3% grade 2 genitourinary toxicity. About half of the patients had grade 1 bowel toxicity and 13% experienced grade 2 bowel toxicity. Only one patient had grade 3 diarrhea during the RT, but this toxicity resolved at a later time point. In terms of the patient-reported outcomes, there was expected worsening of bowel scores at the last RT day that was both statistically and clinically significant when compared to the baseline. Despite this, the patient-reported bowel symptoms improved at 6-week and 3-month follow-up appointments. The early analysis of SPARTACUS suggested that adjuvant stereotactic hypofractionated RT is well tolerated in the post-operative setting of uterine cancers [86]. However, long-term follow-up randomized data and a larger patient population are required to establish SBRT as a potential option in adjuvant pelvic RT.

Following the early encouraging results seen in SPARTACUS, researchers have designed a randomized trial that is currently open and enrolling patients at multiple sites in Ontario, Canada. This trial, entitled SPARTACUS II, randomizes patients between pelvic SBRT with a dose of 30 Gy in 5 fractions and standard fractionation of 45 Gy in 25 fractions over 5 weeks [87]. This phase 2 trial expects to enroll a total of 50 patients with stage I–III uterine cancer. The primary objective of this study is evaluating the EPIC bowel scores between the two randomized groups. There are multiple other secondary objectives of interest, including the bowel and urinary toxicities using CTCAE, urinary impact measured using EPIC, locoregional failure and disease-free survival [87].

Several other trials worldwide are also investigating the role of hypofractionated pelvic radiotherapy in the uterine cancer adjuvant setting. However, SPARTACUS II is the only current trial with a randomized study design to our knowledge. A phase I safety trial from the University of Chicago investigates the most tolerable dose of hypofractionated whole pelvic RT in stage I–III endometrial cancer patients who require adjuvant RT without concurrent chemotherapy or paraaortic RT [88]. This non-randomized trial aims to enroll approximately 40 patients following definitive surgery. The primary outcome is to establish a safe and tolerable dose of hypofractionated whole pelvic radiotherapy during a 3–5-week treatment period. The secondary outcomes include the acute toxicity profile of hypofractionated radiotherapy using the CTCAE scale, GI and GU toxicity post-radiation for 2 years following treatment and pelvic failure rate [88].

Other phase II trials investigating gastrointestinal and urinary toxicity are the RT-PACE trial (A Pilot Study of Adjuvant Hypo-Fractionated Radiotherapy for Non-Metastatic Cervical and Endometrial Cancer) opened by the University of Utah [89] and the PARCERII trial (Postoperative Hypofractionated Radiation in Cervical and Endometrial Tumours: Phase II Study) from the Tata Memorial Centre [90]. Different from SPARTACUS and analogous to RTOG 1203, the patient population in these two trials involves both cervical and endometrial cancer patients undergoing RT post-surgery. The primary aims of these two trials differ. The RT-PACE trial is assessing acute gastrointestinal toxicity in the last week of a 3-week hypofractionated pelvic RT course as the primary objective, while the PARCERII trial will be looking at the late toxicity of moderate hypofractionated EBRT consisting of a dose of 39 Gy in 13 fractions delivered daily over 2.5–3 weeks [89,90].

Another hypofractionated RT trial in endometrial cancers comes from South Korea and is called the Postoperative Hypofractionated Intensity-modulated Radiotherapy Endometrial Cancer: A Prospective Phase II Trial (POHIM_EM Trial). This study has a single-arm design and investigates the rates of disease-free survival in stage III uterine cancer treated with moderate hypofractionated radiotherapy [91]. Despite the narrower inclusion criteria, this trial has a moderate size, and aims to enroll 92 patients with stage III endometrioid endometrial cancer following hysterectomy and surgical staging. Patients will also be treated with moderate hypofractionated IMRT with 2.5 Gy × 16 fractions in the post-operative setting. Finally, another trial focused on the FIGO stage III uterine cancer population is being conducted at Memorial Sloan Kettering Cancer Center. It aims to investigate the feasibility of adjuvant concurrent chemotherapy and short-course RT in stage IIIA and IIIC1 endometrial cancer patients who undergo molecular testing and have copy-number-high or copy-number-low subtypes of endometrial cancer [92]. Different from the other trials, hypofractionated RT is delivered with a reduced dose regimen of 25 Gy delivered in 1 week with concurrent carboplatin and paclitaxel. The aim of the study is to assess the tolerance associated with this adjuvant chemoradiation regimen.

In summary, there are multiple current open trials investigating hypofractionated RT in uterine cancer [87,88,89,90,91,92]. These trials are heterogeneous in design and treatment strategy, with differences in dose fractionation, the population included and the use of concurrent chemotherapy. Nevertheless, these studies will contribute to the body of evidence needed to provide a better understanding of the role of hypofractionated radiotherapy in uterine cancer treatment.

## 5. Conclusions and Future Directions

Hypofractionated radiotherapy is a promising approach to the treatment of gynecologic malignancies. This is particularly true in the context of strained healthcare systems or other low-resource settings and the need to improve global access to radiation therapy. In other pelvic malignancies, the safety and efficacy of hypofractionated RT has been investigated more extensively, and has become the standard of care. In the gynecological cancer space, there is emerging evidence regarding the use of hypofractionation in cervical and uterine cancer treatment with ongoing phase I-II clinical trials in various countries actively exploring the role of hypofractionation. Most of these trials are designed to investigate feasibility, tolerability and early efficacy signals regarding a constellation of different dose fractionation regimens. The success of these trials could potentially result in larger studies aiming to provide definitive answers using collaborative phase 3 randomized trials.

Furthermore, addressing the global disparities in access to radiation therapy remains a critical priority. Initiatives to expand the availability of hypofractionated treatments could help bridge the gap between RT need and availability worldwide. Hypofractionated radiotherapy in gynecologic malignancies has the potential to provide substantial economic benefits by reducing the treatment costs, optimizing resource utilization and improving patient access to care. These cost-effective advantages make hypofractionation an attractive option for both healthcare systems and patients, particularly in resource-limited settings. Hypofractionated RT is not yet a standard-of-care treatment in gynecologic cancers, and further investigation, as discussed in this review, is of utmost importance. However, many patients may remain without RT worldwide while the body of evidence supporting hypofractionated RT in gynecological cancers is still emerging. As such, an open dialogue could be considered by institutions and healthcare providers about the ethics associated with the early adoption of non-standard treatments, like hypofractionated radiotherapy. An argument in favor of this policy stems from the use of hypofractionated radiotherapy as a contingency plan in times of crisis, such as the COVID-19 pandemic, for example. Similarly, the lack of universal access to radiotherapy represents an endemic problem or a “continuing crisis” that results in thousands of preventable deaths worldwide, which may potentially be mitigated by the delivery of hypofractionated RT.

In conclusion, the field of radiation oncology in gynecologic malignancies is currently investigating the use of hypofractionated radiotherapy using multiple ongoing studies. If proven to be successful, these results will lay the foundation for the development of large phase 3 hypofractionation trials that could provide more definitive answers on its role and consolidate its use as a standard-of-care option.

## Data Availability

The data can be shared up on request.
